# A co-culture model to study modulators of tumor immune evasion through scalable arrayed CRISPR-interference screens

**DOI:** 10.3389/fimmu.2024.1444886

**Published:** 2024-10-21

**Authors:** Ramiro Martinez, Chiara Finocchiaro, Louis Delhaye, Fien Gysens, Jasper Anckaert, Wim Trypsteen, Maarten Versteven, Eva Lion, Sandra Van Lint, Karim Vermaelen, Eric James de Bony, Pieter Mestdagh

**Affiliations:** ^1^ OncoRNALab, Center for Medical Genetics (CMGG), Ghent University, Ghent, Belgium; ^2^ Cancer Research Institute Ghent (CRIG), Ghent University, Ghent, Belgium; ^3^ Department of Biomolecular Medicine, Ghent University, Ghent, Belgium; ^4^ Center for Medical Biotechnology, Flanders Institute for Biotechnology – UGENT (VIB-UGENT), Ghent, Belgium; ^5^ Laboratory of Experimental Hematology, Vaccine and Infectious Disease Institute (VAXINFECTIO), Faculty of Medicine and Health Sciences, University of Antwerp, Antwerp, Belgium; ^6^ Tumor Immunology Laboratory, Department of Pulmonary Medicine, Ghent University, Ghent, Belgium

**Keywords:** immuno-oncology, long non-coding RNAs, arrayed screens, CRISPRi, co-culture

## Abstract

Cancer cells effectively evade immune surveillance, not only through the well-known PD-1/PD-L1 pathway but also *via* alternative mechanisms that impair patient response to immune checkpoint inhibitors. We present a novel co-culture model that pairs a reporter T-cell line with different melanoma cell lines that have varying immune evasion characteristics. We developed a scalable high-throughput lentiviral arrayed CRISPR interference (CRISPRi) screening protocol to conduct gene perturbations in both T-cells and melanoma cells, enabling the identification of genes that modulate tumor immune evasion. Our study functionally validates the co-culture model system and demonstrates the performance of the CRISPRi-screening protocol by modulating the expression of known regulators of tumor immunity. Together, our work provides a robust framework for future research aimed at systematically exploring mechanisms of tumor immune evasion.

## 1 Introduction

The field of oncology has seen significant advancements with the advent of immune therapies, revolutionizing the treatment landscape for various cancer types. Among these, immune checkpoint inhibitors targeting CTLA4 or PD-1/PD-L1 have been particularly transformative, improving overall survival rates and establishing durable responses in a subset of treated patients (1). Melanoma is among the tumor types that show the highest response to immune checkpoint inhibition, with response rates up to 61% in patients with unresectable or metastatic melanoma treated with combination immunotherapy (nivolumab and ipilimumab) (2). Nevertheless, a substantial fraction of patients does not respond or fails to achieve long-term remission, underscoring the urgent need to develop novel therapeutic strategies.

The intricate interplay between tumor cells and the immune system is multi-faceted and influenced by an array of molecular and cellular factors that can be modulated by tumor cells to orchestrate immune evasion (3). Mutations that impair or inactivate the functionality of the beta-2-microglobulin (B2M) gene, a crucial component of the MHC class I machinery, or the loss or aberrant expression of MHC class I molecules are well-documented mechanisms of immune evasion (4–6). More recently, CMTM6 and the PD-L1/CD58 axis was discovered as a pivotal element in immune escape, wherein the modulation of these molecules can undermine T-cell mediated cytotoxicity (7). The tumor microenvironment can also harbor immunosuppressive cell populations, including regulatory T-cells (Tregs), myeloid-derived suppressor cells (MDSCs) and M2-type macrophages, creating a milieu conducive to tumor persistence and growth. Within the tumor microenvironment, the competition for nutrients like glucose and lipids can lead to a state of T-cell exhaustion, significantly dampening the anti-tumor immune response (8). The intricate subclonal landscape of tumors like melanoma further complicates the immune evasion narrative, as it contributes to the heterogeneity in immunotherapy response, necessitating a more granular understanding of tumor biology (9).

*In vitro* co-culture systems offer a controlled environment to study interactions between immune cells and tumor cells and can help identify tumor intrinsic factors that modulate immune evasion (10, 11). Gee et al. used a PC9 lung adenocarcinoma cell line genetically modified to express a membrane-bound anti-CD3 antibody to facilitate interaction with CD8+ T cells (12). This system allows to investigate the effect of gene knockdown in tumor cells on T-cell responses, bypassing the need for TCR-mediated specificity to tumor antigens. A notable limitation of this model is the lack of TCR-mediated antigen recognition, which is a critical component of natural tumor-immune interactions. Co-culture models based on patient-derived tumor organoids and T-cells are more physiologically relevant compared to tumor cell lines (13), but are less amenable to high-throughput genetic perturbations. To study the avidity of TCR’s, Morimoto et al. developed the 2D3 cell line, a model derived from Jurkat T-cells that is devoid of an endogenous TCR and engineered to express an NFAT-responsive eGFP reporter as well as constitutive CD8 (14). Activation of the NFAT pathway, a canonical signal transduction cascade downstream of T-cell receptor engagement, drives eGFP expression, providing a quantifiable readout of T-cell activation. Introduction of CD8 stabilizes the interaction between the TCR and the MCH class I molecule, facilitating antigen recognition. The 2D3 cell line was further transduced to express surface programmed death-1 (PD-1), thereby creating a model suitable for assessing T-cell signaling in the context of the PD-1 PD-L1 axis (15).

Here, we present a genetic screening platform in a co-culture of 2D3 cells and melanoma cells that can be applied to identify modulators of tumor immune evasion. We developed a workflow for arrayed lentiviral delivery of single guide RNAs (sgRNA) that does not require bacterial transformation, enabling high-throughput perturbation of gene expression in a co-culture setup. Through knock down of known modulators of tumor immunogenicity, we demonstrate the functionality of this platform and highlight its potential to uncover novel modulators of immune evasion.

## 2 Materials and methods

### 2.1 Cell culture

The embryonic kidney cell line HEK293T/17 (ATCC, Cat. #CRL-11268), the malignant melanoma cell lines MALME-3M (ATCC, Cat. #HTB64) and SK-MEL-5 (ATCC, Cat. #HTB-70), the 2D3 cell line, a Jurkat derivative, and antigen presenting cell lines (APCs) T2 (174 x CEM.T2, ATCC, Cat. #CRL-1992), and U266B1 (ATCC, Cat. #TIB-196) were employed in the study.

MALME-3M and T2 cells were cultured in Iscove’s Modified Dulbecco’s Medium (IMDM; Thermo Fisher Scientific, Cat. #12440-053) supplemented with 20% fetal bovine serum (FBS; Merck Life Science, Cat. #F0804-500ML) and 100 U/mL penicillin and 100 ug/mL streptomycin (1%, PS). The 2D3 and U266B1 lines were maintained in Roswell Park Memorial Institute Medium (RPMI-1640; Thermo Fisher Scientific, Cat. #11875-093) with 10% FBS and 15% FBS respectively, each supplemented with 1% PS. HEK293T/17 cells were cultured in Dulbecco’s Modified Eagle’s Medium (DMEM; Thermo Fisher Scientific, Cat. #11965-092) with high glucose, supplemented with 10% FBS and 1% PS. SK-MEL-5 cells were cultured in Eagle’s Minimum Essential Medium (MEM; GIBCO. #12599049) supplemented with 10% FBS, 1 mM sodium pyruvate (Thermo Fisher Scientific, Cat. #11360070), 1% PS and 1% L-Glutamine.

All cell lines were incubated at 37°C in a 5% CO_2_ humidified environment. Adherent cell lines, including HEK293T/17, MALME-3M, and SK-MEL-5, were passaged every 3 days or when reaching 80-90% confluence, whereas the suspension cell lines 2D3, T2, and U266B1 were kept between 3 x 10^5 and 1 x 10^6 viable cells/mL. Adherent cells were detached using 0.05% Trypsin-EDTA (Thermo Fisher Scientific, Cat. #25300062) for 3-5 minutes at 37°C and neutralized with an equal volume of complete medium before reseeding at appropriate densities. Cells were tested every month for mycoplasma contamination using the MycoAlert™ Mycoplasma Detection Kit (Lonza, Cat. #LT07-318) according to the manufacturer’s instructions and were authenticated every 6 months by STR genotyping using the GenePrint^®^ 10 System (Promega, Cat. #B9510).

### 2.2 Digestion of the pSLQ1371 plasmid

The plasmid pSLQ1371 (16) (Addgene #60955) is designed for sgRNA expression with a U6 promoter and includes puromycin resistance and BFP (blue fluorescent protein) for tracking. It was subjected to a two-step digestion process using an optimized protocol developed in our laboratory. In the initial phase, 5 µg of plasmid was mixed with 10 units of BstX1 enzyme (NEB, Cat. #R0113L) and 5 µL of 10X Buffer 3.1 (NEB, Cat. #B7203S), and nuclease-free water to a final volume of 50 µL for each reaction. This mixture was incubated at 37°C for 3 hours. When scaling the reaction to accommodate more plasmid, all components—including DNA, enzyme, and buffer volumes—were increased proportionally to maintain consistent conditions. Reactions were then combined, and the buffer and enzyme were removed using DNA purification columns (ZymoResearch, Cat. #D4031) fallowing manufacturers recommendation. Notably, each column, designed to handle 500 µg, was used at 80% of its maximum capacity to prevent saturation of the column and loss of DNA.

Next, 5 µg of the digested and purified plasmid was combined with 10 units of BlpI enzyme (NEB, Cat. #R0585L), 10 units of phosphatase Quick CIP (NEB, Cat. #M0525L), and 5 µL of 10X CutSmart buffer (NEB, Cat. #B7204S), and nuclease-free water to a final volume of 50 µL for each reaction. This mixture was incubated again at 37°C for 3 hours. Subsequently, the doubly digested and phosphatase-treated DNA was subjected to another round of purification using the DNA purification columns. The final DNA product’s purity, assessed with a NanoDrop-1000, was considered pure with an A260/A280 ratio between 1.8 and 2.0 and an A260/A230 ratio greater than 2.0. Quantity was determined using a Qubit Fluorometer with the dsDNA HS Assay Kit (Thermo Fisher Scientific, Cat. #Q32851), according to the manufacturer’s protocol.

### 2.3 Lentiviral production and transductions of dCas9 machinery and TCR

HEK293T/17 cells were seeded at 8 x 10^6 cells in 15 mL DMEM complete per T75 flask, targeting ~90% confluency for transfection. Utilizing a second-generation lentiviral packaging system, the following plasmids were transfected: 2 µg of pCMV-dR8.2 (Addgene, Cat. #8455), 8 µg of pCMV-VSV-G (Addgene, Cat. #8454), and 10 µg of pLV-EF1a-3XFLAG-NLS-dCas9-G4S-KRAB-MeCP2_mPGK-NeoR [generated with an in-house developed Golden Gate assembly system (17)]. For transfection, plasmids were added to 1.5 mL of Opti-MEM (Fisher Scientific, Cat. #31985070) and 60 µL of TransIT-Lenti reagent (Mirus, Cat. #MIR6600) at a 3:1 (w:v) DNA/reagent ratio, and the solution was gently mixed by pipetting. After 10 minutes of incubation at room temperature to allow complex formation, the mixture was added dropwise to the HEK293T/17 cells ensuring even distribution. Supernatant containing lentivirus was harvested at 48 hours post-transfection, carefully collected to avoid disturbing the cells, centrifuged (568 g, 5 minutes) to remove cell debris, filtered (0.45-micron; Merck, Cat. #HVLP06225), aliquoted (1.5 mL/cryovial), and stored at -80°C.

For dCas9-KRAB-MeCP2 integration into target cells, MALME-3M and SK-MEL-5 cells were seeded at a density of 4 x 10^5 cells/well, while 2D3 cells at a density of 1 x 10^6 cells/well in 6-well plates. 24 hours later, the cell culture media for each cell line was removed, and each well was supplemented with 1.5 mL of viral supernatant. For the 2D3 and SK-MEL-5, 5 µg/mL of polybrene was added to the viral supernatant to enhance viral uptake. This step was omitted for the MALME-3M cell line due to observed cytotoxicity. This was followed by spinoculation at 568 g at 32°C for 2 hours. After spinoculation, 3.5 mL of fresh complete culture medium was added to each well. The cells were then incubated for an additional 48 hours to enhance viral integration. Post-infection, cells were transferred to T25 flasks with fresh media. Non-transduced control cells were maintained in parallel to assess transduction efficiency. The day after, infected cells were selected by adding 600 µg/mL of geneticin. This selection pressure was maintained until complete elimination of the control cells was achieved (between 7-15 days). For the maintenance of the stably transduced cell populations, the geneticin concentration was subsequently reduced to 300 µg/mL.

The transduction of 2D3^dCas9^ cells with MART1-TCR followed the virus production and infection procedure previously described, using the vector pLV-EF1a-MART1-TCR-PGK-Hygro (17). Selection was carried out using 500 µg/mL hygromycin for 12 days, followed by a maintenance dose of 200 µg/mL.

### 2.4 Western blot analysis

A 6-well plate was prepared by seeding each well with a density of 3 x 10^5 cells in 2 mL of correspondent complete medium. The configuration was as follows: SK-MEL-5-dCas9-KRAB-MeCP2 (SK-MEL-5^dCas9^) cells were seeded in an untreated well and another treated with IFN-γ (Genaxxon, Cat. #C6018.1000) at a concentration of 150 ng/mL. MALME-3M-dCas9-KRAB-MeCP2 (MALME-3M^dCas9^) and MALME-3M-dCas9-KRAB-MeCP2 PD-1-KnockDown cells were both seeded in one untreated well and in another treated with IFN-γ at a concentration of 200 ng/mL. In the case of 2D3^TCR/dCas9^ cells, we used 1 x 10^6 cells per mL.

Proteins were extracted from every well 24 hours later by collecting the cell pellets. Pellets were lysed using RIPA buffer (40.5 mL distilled water, 250 mg sodium deoxycholate, 1.5 mL of a 5 M NaCl solution, 2.5 mL of a 1 M Tris-HCl solution pH 7.5, 0.5 mL of a 10% SDS solution, and 5 mL of a 10% NP-40 solution) supplemented with protease inhibitors (Complete Mini EDTA-free Protease Inhibitor Cocktail Tablets, Roche, Cat. #11836170001) for 30 min at 4°C. Samples were vortexed every 10 minutes to ensure complete lysis. Lysates were then clarified *via* centrifugation at 10,000 x g for 15 min at 4°C. Protein concentrations were determined using a BCA assay (Pierce™ BCA Protein Assay Kit, Thermo Fisher Scientific, Cat. #23225), with absorbance readings taken on a GloMax Discover spectrophotometer against a BSA-derived standard curve. Equal amounts of protein (50 μg) were heated at 95°C for 5 min to ensure complete denaturation, combined with 1X Laemmli buffer (62.5 mM Tris-HCl pH 6.8, 2% SDS, 10% glycerol, 0.01% bromophenol blue, and 5% β-mercaptoethanol), loaded into 10% polyacrylamide gels, and separated at 100 V for an hour. Subsequent protein transfer to nitrocellulose membranes was conducted at 100 V for an hour using a wet transfer system with transfer buffer (25 mM Tris base, 192 mM glycine, 20% methanol).

After an hour-long blocking step in 5% milk (non-fat dry milk powder) in Tris-buffered saline with Tween 20 (TBST; 20 mM Tris-HCl pH 7.6, 150 mM NaCl, 0.1% Tween 20), membranes were incubated with appropriate primary antibodies overnight at 4°C with gentle shaking (approximately 30 rpm). dCas9 expression was assessed with mouse monoclonal anti-Cas9 antibody (Diagenode, Cat. #C15200203-100) at a dilution of 1:4000, and mouse monoclonal anti-β-actin antibody (ThermoFisher, Cat. #A2228) at a dilution of 1:5000 as a loading control. For PD-L1 and MART1 protein level evaluation, mouse monoclonal anti-PD-L1 antibody (CellSignaling, Cat. #29122T) and rabbit monoclonal anti-MART1 antibody (Cell Signaling, Cat. #64718) were used at dilutions of 1:1000. Concurrently, rabbit monoclonal anti-vinculin antibody (CellSignaling, Cat. #E1E9V) was used at a dilution of 1:1000 as a loading control for both assays.

Following primary antibody incubation, blots were washed three times for 5 minutes each with TBST. Blots were then exposed to secondary antibodies: Anti-Mouse IgG HRP (Vector Laboratories, Cat. #PI-2000, 1:10.000) or Anti-Rabbit IgG HRP (Vector Laboratories, Cat. #PI-1000, 1:10.000) as required for 1 h at room temperature with gentle shaking. Chemiluminescent signals were produced using the Supersignal™ West Femto Substrate (Thermo Scientific, Cat. #34095) and captured on an Amersham Imager 680.

### 2.5 Knockdown efficiency validation

MALME-3M^dCas9^, SK-MEL-5^dCas9^, and 2D3^TCR/dCas9^ cell lines were cultured in 96-well plates with seeding densities of 5 x 10^3 cells per well for SK-MEL-5^dCas9^ and MALME-3M^dCas9^, and 15 x 10^3 cells per well for 2D3^TCR/dCas9^, using 100 µL of respective growth medium. The subsequent day, cells underwent spinoculation performed at 568 g and 32°C for 2 hours with 100 µL of lentiviral supernatant containing sgRNAs targeting NEAT1, DPH1, PD-L1 (only MALME-3M^dCas9^ cells), PD-1 (only for 2D3^TCR/dCas9^), or non-targeting sgRNAs aimed at genomic desert areas as controls. For 2D3^TCR/dCas9^ cells, 5 µg/mL polybrene was included to facilitate viral transduction. Post-spinoculation, an additional 100 µL of fresh medium was added to each well, bringing the total volume to 200 µL. After 48 hours, each medium was replaced with 200 µL containing 1 µg/mL Puromycin to remove untransduced cells. Forty-eight hours later medium was refreshed with complete growth medium to allow cells to recover. For MALME-3M cells, 24 hours post-recovery, the medium was replaced with 200 µL containing 200 ng/mL IFN-γ to induce PD-L1 expression for the knockdown assay.

Total RNA extraction was performed using the SingleShot™ Cell Lysis Kit (Bio-Rad, Cat. #1725080) according to the manufacturer’s protocol. cDNA synthesis was carried out using the iScript™ cDNA Synthesis Kit (Bio-Rad, Cat. #1708890) according to the manufacturer’s protocol. qPCR was conducted using the SsoAdvanced™ Universal SYBR^®^ Green Supermix (Bio-Rad, Cat. #1725274) in 5 µL reactions containing 2.5 µL of SYBR Green Supermix, 0.25 µL each of forward and reverse primers (5 µM), and 2 µL of cDNA. Cycling conditions were 95°C for 3 minutes, followed by 40 cycles of 95°C for 10 seconds and 60°C for 30 seconds. Sequences of primers and sgRNA used for NEAT1, DPH1, PD-L1, and reference genes can be found in **Supplementary Table 1**.

### 2.6 Validation of TCR^MART1^ with peptide pulsed APC

T2 and U266B1 antigen presenting cells were employed alongside the 2D3^TCR/dCas9^ T cell line model. Peptide pulsing on APCs was performed by incubating cells at a concentration of 2 x 10^6 cells per mL with MART1 antigen peptide [Leu27] - (26-35) - ELAGIGILTV (Genaxxon, Cat. #P2508.9501), which represents a single epitope, at concentrations of 1, 5, 25 µg/mL for T2, and 5, 25, 50 µg/mL for U266B1. The pulsing procedure involved a 1-hour incubation at room temperature with continuous rolling on a tube rotator at 20 rpm, followed by centrifugation at 300 x g for 5 min and resuspension in fresh media to remove excess peptide.

Two 96-well plates were prepared for the co-culture assays. One plate was designated for the 2D3^TCR/dCas9^ and U266B1 co-culture, and the other for 2D3^TCR/dCas9^ and T2 co-culture. Co-cultures were established at ratios of 100:1, 10:1, 3:1, 1:1, and 1:2 (2D3^TCR/dCas9^:APC). Each well contained a fixed cell count of 2 x 10^5, allocated according to the specified ratios. For instance, a 1:1 ratio entailed 1 x 10^5 2D3^TCR/dCas9^ cells co-cultured with 1 x 10^5 APCs.

For reference controls, two distinct setups were employed. The negative control consisted of 2D3^TCR/dCas9^ T cells co-cultured with non-peptide-pulsed APCs at a 2:1 ratio, specifically designed to evaluate baseline T-cell activation without antigenic stimulation. In contrast, the positive control involved monocultures of 2D3^TCR/dCas9^ treated with 25 ng/mL of Phorbol Myristate Acetate (PMA) (*In vivo*Gen, Cat. #tlrl-pma) and 1 µg/mL of Ionomycin (CellSignalling, Cat. #9995S), to ensure maximum stimulation as a benchmark for T-cell activation.

Post 24-hour co-culture, cells were stained following manufacturer recommendations with a CD8a-specific antibody to differentiate 2D3^TCR/dCas9^ cells from APCs. Briefly, cells were transferred to flow cytometry tubes, washed once with PBS containing 2% FBS, and incubated with anti-human CD8a-APC antibody (BioLegend, Cat. #301049) at a dilution of 1:100 in 100 µL staining buffer for 30 minutes at 4°C in the dark. After staining, cells were washed twice with staining buffer and resuspended in 200 µL of PBS for analysis. The FORTESSA-X20 cytometer plate reader was employed to quantify the percentage of eGFP positive cells, indicating the level of T-cell activation in response to APC interaction. Data acquisition involved collecting 10,000 events per sample, with eGFP detected in the FITC channel and CD8a in the APC channel. Results from all co-culture conditions are presented in **Supplementary Figure 1**.

### 2.7 Methodology for co-culture model component validation on tumor cells

MALME-3M^dCas9^ cells (2.5 x 10^4 cells/well) were cultured in 96-well plates in 200 µL of complete IMDM media. A subset of these wells was subjected to a 24-hour treatment with 200 ng/mL of IFN-γ.

24hs later, the media was removed to eliminate residual IFN-γ. 2D3^TCR/dCas9^ cells were then added to both IFN-γ-treated and untreated MALME-3M^dCas9^ wells at varying ratios: 1:1, 1:2, 1:3, 1:4, and 1:5, leading to the incorporation of 3 x 10^4, 6 x 10^4, 9 x 10^4, 12 x 10^4, and 15 x 10^4 2D3^TCR/dCas9^ cells, respectively (adjusted to maintain a total volume of 200 µL per well). A subset of these co-cultures was then treated with 15 µg/mL of nivolumab. For reference controls, 2D3^TCR/dCas9^ monocultures were established. One set remained untreated, delineating the negative control for T-cell activation, whereas another set was exposed to 25 ng/mL of PMA (*In vivo*Gen, Cat. #tlrl-pma) and 1 µg/mL of Ionomycin (Cell Signaling, Cat. #9995S), establishing the positive control.

Twenty-four hours later, eGFP fluorometric quantification was carried out across all wells using the FORTESSA-X20 cytometer plate reader. Results from all co-culture conditions are presented in **Supplementary Figure 2**.

### 2.8 Stepwise protocol: arrayed screen on MALME-3M – high-throughput sgRNA lentiviral production

#### 2.8.1 Step 1: Design of sgRNAs

- Utilize the CRISPICK tool (18, 19) from the Broad Institute (https://portals.broadinstitute.org/gppx/crispick/public) to design sgRNAs targeting specific genes of interest.
- Identify the transcription start site (TSS) of the most expressed transcript for each target gene by analyzing RNA sequencing data produced in this study. These data are available in the GEO database under the accession number GSE269453.
- Input gene chromosome location, strand orientation, and TSS start site into the design tool. The settings used for the design in this study were:
  - Reference genome: Human GRCh38 (Ensembl v.112)
  - Mechanism: CRISPR interference (CRISPRi)
  - Enzyme: Spy dCas9-KRAB-MeCP2 (Chen et al., 2013)
- Obtain four sgRNA sequences per gene.
- Design the antisense sequences by deriving the reverse complement from the original sgRNA.
- Modify sgRNA sequences to incorporate sticky ends compatible with the BstX1 and BlpI digested pSLQ1371 vector (see **Supplementary Table 1** for sequences).
- Sense and antisense components of the sgRNA guides were procured from IDT in 96 well plates (both components in the same well per gene) at normalized concentration of 10 nm. For each gene target, four sgRNAs were designed. Two sgRNAs were placed in separate wells of one plate, and the other two in an identically positioned wells on a second plate, facilitating post-annealing pooling.

#### 2.8.2 Step 2: Preparation of oligonucleotides (Day 1)

- Resuspend oligonucleotides to a final concentration of 100 µM by adding 100 µL of nuclease-free water.
- In two new 96 well plates add:
  - 4 µL of 100 µM sense and antisense oligo mix
  - 1 µL of 10X T4 DNA Ligase Buffer (NEB, Cat. #B0202S)
  - 5 µL of nuclease-free water
- Subject the mixture to annealing in a thermocycler:
  - 95°C for 3 minutes
  - Gradual cooling to 12°C at 0.1°C per second
- Store annealed oligos at -20°C until further use.
*Note: Pre-pooling of sgRNA sense and antisense oligos prior to the annealing step is avoided to prevent the formation of nonspecific annealed products.*

#### 2.8.3 Step 3: Phosphorylation of annealed oligos (Day 1)

- Combine 5 µL of annealed oligos from the two plates in pairs to obtain two sgRNAs per gene per well in a final volume of 10 µL.
- To each combined well, add:
  - 1 µL of T4 Polynucleotide Kinase (10 units/µL, NEB, Cat. #M0201S)
  - 4 µL of 10X T4 DNA Ligase Buffer
  - Nuclease-free water to a final volume of 50 µL
- Incubate at 37°C for 1 hour in a thermocycler.
- Inactivate the enzyme at 65°C for 20 minutes.

#### 2.8.4 Step 4: Ligation into pSLQ1371 vector (Day 1)

- Prepare the ligation reaction using half-reactions, as found effective for this protocol, by mixing:
  - 225 ng of BstX1 and BlpI digested pSLQ1371 vector (prepared as described in section 2.2)
  - Diluted phosphorylated oligos to maintain a vector-to-insert molar ratio of 1:20 (approximately 20 ng of oligo insert)
  - 0.5 µL of T4 DNA Ligase (NEB, Cat. #M0202S)
  - 1 µL of 10X T4 DNA Ligase Buffer
  - Nuclease-free water to a final volume of 10 µL
- Incubate the ligation mixture at 16°C overnight (approximately 16-18 hours).

#### 2.8.5 Step 5: Preparation for transfection

- No purification step is necessary post-ligation. Proceed directly to transfection.

#### 2.8.6 Step 6: Lentiviral production (Day 2)

- Seed HEK293T/17 cells at 4.2 x 10^4 cells per well in 110 µL of complete DMEM in Nunc™ Edge™ 96-Well, Nunclon Delta-Treated, Flat-Bottom Microplates (Thermo Fisher Scientific, Cat. #167574) to mitigate edge effects. Add 1.5mL of sterile water to each reservoir of the plate. Incubate overnight to reach ~90% confluency.
- Prepare two master mixes for transfection (Scale as needed. Working solutions of packaging and envelope plasmids are 1 µg/µL):
  - Master Mix 1 (per ligation-mix-well):
    ▪ 180 ng of pCMV-dR8.2 (Addgene, Cat. #8455)
    ▪ 45 ng of pCMV-VSV-G (Addgene, Cat. #8454)
    ▪ 27.9 µL of Opti-MEM (Thermo Fisher Scientific, Cat. #31985070)
  - Master Mix 2 (per well):
    ▪ 1.35 µL of TransIT-Lenti reagent (Mirus, Cat. #MIR6600)
    ▪ 27.9 µL of Opti-MEM
*NOTE: Master Mix 2 should be prepared just before use to minimize premature complex formation without the cloned vector, ensuring optimal transfection efficiency.*

- Add 28 µL of Master Mix 1 to each well containing the ligation product. Mix thoroughly by pipetting up and down 10 times.
- Add 29 µL of Master Mix 2 to each well. Pipette up and down 10 times to ensure proper mixing.
- Incubate at room temperature for 10 minutes to allow complex formation.
- Add gently 21 µL of the transfection mixture to each well of the HEK293T/17 cell plate in triplicates. Shake the plate gently in a circular pattern to promote even distribution of complexes.
- Seed MALME-3M^dCas9^ cells at 4 x 10^3 cells per well in 200 µL of complete IMDM in a 96-well plate one day prior to infection.

#### 2.8.7 Step 7: Viral harvesting and infection (Day 4)

- Forty-eight hours post-transfection, collect the viral supernatant from each well.
- Filter the supernatant using a MultiScreen HTS GV Filter Plate, 0.22 µm (Millipore, Cat. #MSGVS2210) fallowing manufacturers recommendation.
- Pool the filtered supernatants from triplicates to ensure equal viral titers for triplicates.
- Carefully aspirate and discard the existing 200 µL of media from each well of the MALME-3M^dCas9^ cell plate. Replace it with 110 µL of the viral supernatant to initiate infection.
- Perform spinoculation by centrifuging the plate at 568 g and 32°C for 2 hours.
- After spinoculation, add 100 µL of fresh complete IMDM on top of the viral supernatant.

#### 2.8.8 Step 8: Selection of transduced cells (Day 6)

- Forty-eight hours post-infection, begin selection by replacing the media with 200 µL of complete IMDM containing 1 µg/mL puromycin.
- Monitor cell viability daily, and once non-infected control cells have perished (approximately 72 hours post-infection), switch to puromycin-free IMDM to allow cell recovery.

#### 2.8.9 Step 9: Induction of PD-L1 expression (Day 10)

- Once cells reach 80-90% confluency (48hs-72hs post-selection), replace the media with 200 µL of fresh complete IMDM containing 200 ng/mL of IFN-γ.
- Incubate for 24 hours to induce PD-L1 expression.

#### 2.8.10 Step 10: Co-culture with 2D3TCR/dCas9 cells (Day 11)

- Remove the media containing IFN-γ and gently wash the cells with PBS to remove residual cytokine.
- Add 6 x 10^4 2D3^TCR/dCas9^ cells in 200 µL of RPMI complete media to each well, establishing an approximate 2:1 ratio of T cells to MALME-3M^dCas9^ cells.
- Incubate the co-cultures for 24 hours at 37°C with 5% CO_2_.

#### 2.8.11 Step 11: eGFP signal quantification (Day 12)

- Measure eGFP fluorescence using the Fortessa-X20 plate reader.
- Set the sample intake speed to 1.5 µL/sec and perform 3 mixes per well prior to reading to ensure homogeneity and detachment of 2D3^TCR/dCas9^ cells from MALME-3M^dCas9^.

### 2.9 Quantification of ligation efficiency using droplet digital PCR

For the QX200 ddPCR (Bio-Rad, Cat #186-4033) assay, the reaction mixture was composed of 10 µL ddPCR EvaGreen supermix, 8 µL nuclease free water, 0.5 µL of each 10 µM forward and reverse primers (**Supplementary Table 1**), and 1 µL of the previously digested ligation mix (7 x 10^6 x dilution), leading to a total volume of 20 µL. Following droplet generation, the samples were subjected to PCR amplification under the following conditions: an initial activation step at 95°C for 5 minutes, followed by 40 cycles of denaturation at 96°C for 30 seconds and annealing at 58°C for 1 minute. This was followed by signal stabilization steps at 4°C for 5 minutes and 90°C for 5 minutes. The samples were then held at 12°C indefinitely. Post amplification, population counts and quantification were determined<i> via a QX200 droplet reader and the Quantasoft software (1.7.4.0917, Bio-Rad). All samples were analyzed in duplicate.

### 2.10 Cytotoxicity assay of bulk PBL against SK-MEL-5^dCas9/eGFP^ and MALME-3M^dCas9/eGFP^ melanoma cells

#### 2.10.1 Transduction of melanoma cells with eGFP

MALME-3M^dCas9^ and SK-MEL-5^dCas9^ cells were transduced with a pLV-eGFP-Puromycin vector (17), following the transduction protocol previously described in section 2.3. After transduction, cells were selected with 1 µg/mL puromycin for 72 hours until non-transduced control cells were eliminated. The puromycin concentration was then reduced to a maintenance level of 0.2 µg/mL.

#### 2.10.2 Generation of MART1 mRNA

The MART1 antigen sequence was synthesized as a gBlock (Integrated DNA Technologies, IDT) and cloned into an mRNA vector backbone using the Gibson Assembly method (New England Biolabs, Cat. #E2611S). Competent cells were transformed with the assembled plasmid, followed by plasmid DNA purification using the QIAprep Spin Miniprep Kit (Qiagen, Cat. #27106). Quality control included Sanger sequencing and agarose gel electrophoresis after digestion with specific restriction enzymes. DNA concentration and purity were measured using a NanoDrop ND-1000 spectrophotometer (Thermo Fisher Scientific). The plasmid was linearized with BspQI restriction enzyme (NEB, Cat. #R0712S) for *in vitro* transcription.

*In vitro* transcription of mRNA was performed using the mMESSAGE mMACHINE^®^ T7 Ultra Kit (Thermo Fisher Scientific, Cat. #AM1345) according to the manufacturer’s instructions. RNA concentration and purity were determined with the NanoDrop ND-1000 spectrophotometer, and RNA integrity was assessed using a Bioanalyzer 2100 (Agilent Technologies, Cat. #G2939BA).

#### 2.10.3 Monocyte-derived autologous dendritic cell culture

Monocyte-derived dendritic cells were generated as previously described (Brabants et al., 2018) (20). Briefly, peripheral blood mononuclear cells (PBMC) from anonymous healthy HLA-A2+ donors are separated into monocytes (CD14+ fraction) and peripheral blood lymphocytes (PBL) using immunomagnetic separation (MACS) with anti-CD14 microbeads, according to the manufacturer’s instruction (Miltenyi). After separation, PBLs were frozen in RPMI containing 20% Alburex20 (Human serum Albumin 20 g/l) and 10% dimethyl sulfoxide. CD14+ monocytes were cultured in GMP cell differentiation bags at a density of 1×106 cells/ml in serum-free GMP CellGro medium containing 1000 U/ml pharmaceutical-grade granulocyte macrophage colony-stimulating factor (GM-CSF) (Leukine sargramostim), 500 U/ml GMP-certified recombinant human interleukine-4 (hulL-4). On day 3 of the culture, 2,5 mg/ml synthetic TLR4 agonist Monophosphoryl lipid A (MPLA) and 1000 IU/ml pharmaceutical-grade IFN-y (Immukine) were added to the culture medium for another 24h. Mature DCs (mDCs) were harvested on day 4.

#### 2.10.4 Electroporation of dendritic cells

Cells were harvested and washed twice with Opti-MEM prior to electroporation. DCs were resuspended in Opti-MEM and transferred to 4 mm gap electroporation cuvettes (Bio-Rad, Cat. #1652088). Nuclease-free water containing MART1 mRNA at 1µg per 10^6^ cells was added to the cell suspension. Electroporation with nuclease-free water (MOCK DC) served as a negative control. Electroporation was performed using the Gene Pulser Xcell Electroporation System (Bio-Rad, Cat. #1652660) with a square-wave pulse of 500 V for 1 millisecond. Immediately after electroporation, DCs were transferred to CellGro DC medium containing 1000 U/mL GM-CSF and 250 U/mL IL-4. Cells were cultured in GMP cell differentiation bags at a density of 1 × 10^6^ DCs/mL and incubated for 4 hours at 37°C and 5% CO_2_. After 4 hours, DC phenotype and electroporation efficiency were evaluated using flow cytometry. DCs were then cryopreserved in a medium containing 10% DMSO.

#### 2.10.5 Flow cytometry analysis

To perform surface staining, cells were washed and resuspended in phosphate buffered saline (PBS) supplemented with 0,5 mM ethylenediaminetetraacetic acid (EDTA); 0,25% bovine serum albumin (BSA); and 0,05% sodium azide (NaN3) further referred to as flow cytometry buffer. To prevent non-specific binding, cells were pre-incubated for 30 min at 4°C with anti-human FcR-blocking reagent after which cells were washed with flow cytometry buffer. To identify dead cells, a fixable viability eFluor506 dye was used.

Surface staining of PBMC, PBL and CD14+ fraction after ferromagnetic isolation was performed by staining for 30 min at 4°C with a cocktail of following fluorochrome-conjugated anti-human monoclonal antibodies: anti-CD14-FITC; anti-CD3-BV421; anti-CD19-PE-Cy7 and anti-CD56-PE after which cells were washed and resuspended in flow cytometry buffer.

Dendritic cell phenotype was evaluated by staining for 30 min at 4°C with following anti-human antibody cocktails: anti-CD11c-APC (clone S-HCL-3),; anti-HLA-DR-APC-Cy7 (clone L243) supplemented with anti-CD40-PE (clone SC3); antiCD80-PE (clone 2D10.4),; anti-CD83-PE (clone HB15c); CD86-PE (clone IT2.2),; anti-CD70-PE (clone REA292); anti-CD274-PE (clone MIH1) or anti-CCR7-PE (clone REA108).

Samples were acquired on a Fortessa LSR and analyzed using FlowJo software (Version 10).

#### 2.10.6 *In vitro* priming of T cells

Mock-electroporated or MART1 mRNA-loaded DCs were used to prime naïve autologous T cells *in vitro*. PBLs were thawed and co-cultured with DCs at a ratio of 10:1 (PBLs) in RPMI-1640 medium supplemented with 10% human AB serum (Sigma-Aldrich, Cat. #H4522), 100 U/mL penicillin, and 100 µg/mL streptomycin. To support T cell fitness, recombinant human IL-2 (PeproTech, Cat. #200-02) was added to the culture. Two rounds of *in vitro* stimulation were performed on day 0 and day 7 by adding fresh DCs to the T cell culture at the same ratio. On day 14, stimulated T cells were harvested and used as effector cells for the cytotoxicity assay.

#### 2.10.7 Cytotoxicity assay

SK-MEL-5^dCas9/eGFP^ and MALME-3M^dCas9/eGFP^ cell lines were seeded in 96-well plates at a density of 5 × 10³ cells per well in 100 µL of their respective complete media.

Bulk PBL T cells, either MART1-primed or MOCK-treated, were added at 1 × 10^4^ cells per well in 100 µL of RPMI-1640 complete media, achieving an effector-to-target ratio of 2:1 and a final volume of 200 µL. The plates were placed in an Incucyte S3 Live-Cell Analysis System (Sartorius) to capture hourly images over a 48-hour period using phase-contrast and green fluorescence channels. Cell confluence and eGFP intensity per area were analyzed using Incucyte software to assess cytotoxicity. The eGFP fluorescence by area, normalized to measurements at the 0-hour time point, served as a readout in our analyses.

All conditions were tested in triplicate to allow statistical evaluation. Control wells containing only tumor cells or only PBL T cells were included to account for background signals and spontaneous cell death.

### 2.11 Statistics & data analysis

All statistical analyses were performed using R software. Data is presented as mean values ± standard deviation (SD) from triplicate measurements. The significance of differences between groups was assessed using a two-tailed Student’s t-test, accompanied by the Benjamini-Hochberg procedure. A p-value of less than 0.05 was considered statistically significant, denoted as * in the figures.

Graphical representations of the data were created using R (21). Bar plots were generated using the *ggplot2* package (version 3.4.4), and heatmaps were designed using the *pheatmap* package (version 1.0.12).

## 3 Results

### 3.1 Validating T-cell activation in a co-culture setup

**Figure 1:**
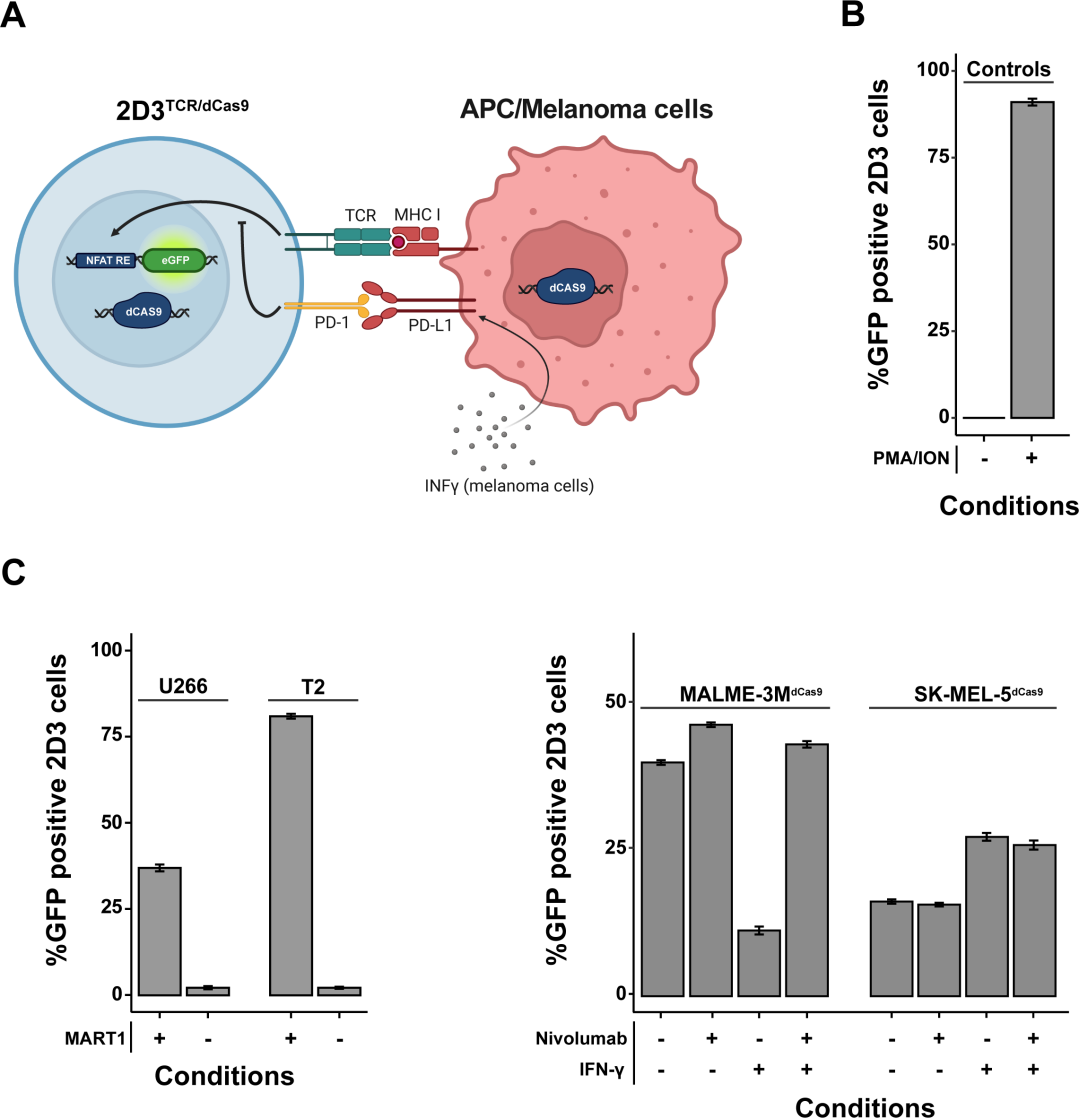
*In Vitro* Validation of a Genetically Engineered T-Cell Activation Model. **(A)** Schematic representation of the genetically modified Jurkat T-cell line designed to report T-cell activation *via* NFAT-driven eGFP expression. These cells are equipped with a TCR specific for HLA-A*02-restricted MART1, CD8 co-receptor, PD-1 receptor, and a dCas9-KRAB-MeCP2 transcriptional repression system. When co-cultured with APCs/melanoma cells expressing MART1, eGFP expression indicates robust T-cell activation. In certain melanoma cell lines, robust expression of surface PD-L1 can be achieved by pre-treatment for 24 hours with IFN-γ. **(B)** Validation of the NFAT-driven eGFP reporter functionality in the T-cell model using flow cytometry. Negligible T-cell activation is observed in the absence of antigen-presenting or tumor cells (quiescent state), while treatment with PMA and ionomycin results in near-total activation. **(C)** Comparative analysis of T-cell activation following co-culture with different antigen-presenting cells (APCs) pulsed with MART1 peptides and melanoma cell lines. 2D3s were co-cultured at a 1:2 tumor cell/APC to 2D3^TCR/dCas9^ ratio for 20-24 hours. The 2D3^TCR/dCas9^ T-cell line exhibits moderate activation with peptide-pulsed U266-B1 cells and pronounced activation with peptide-pulsed T2 cells. Negative controls with non-peptide-pulsed APCs confirm assay specificity. As for melanoma cell lines, additional examination of checkpoint blockade with nivolumab and IFN-γ treatment effects on co-culture-mediated T-cell activation demonstrates distinct immune evasion mechanisms. All data shown represent the mean values of triplicate measurements, with error bars indicating the standard deviation.

To build a stable and robust model to study modulators of T-cell activation in co-culture systems, we transduced 2D3 cells with an HLA-A*02-restricted TCR for the melanoma-associated antigen MART1 and the dCas9-KRAB-MeCP2 transcriptional repression system (**Figure 1A**). We will further refer to these cells as 2D3^TCR/dCas9^. Functionality of the NFAT-driven eGFP reporter in the 2D3^TCR/dCas9^ cells was validated by treatment with PMA and ionomycin, which activate the NFAT pathway in a TCR-independent manner. Flow cytometry analysis revealed 91.0% (95% CI 90.0%–92.0%) eGFP positive 2D3^TCR/dCas9^ cells upon PMA/ionomycin treatment, confirming retainment of the NFAT eGFP reporter functionality in the engineered cell line (**Figure 1B**). When 2D3^TCR/dCas9^ cells were co-cultured with antigen-presenting T2 or U266-B1 cells pulsed with MART1 peptide, we observed robust activation of the T-cells, as evidenced by increased eGFP expression compared to the non-peptide pulsed conditions (**Figure 1C**, left). Co-culture with peptide-pulsed U266-B1 or T2 cells resulted in 36.9% (95% CI 36.7%–37.2%) or 81.0% (95% CI 80.4%–81.6%) of eGFP-positive 2D3^TCR/dCas9^ cells respectively, compared to 2% (95% CI 1.9%–2.1%) eGFP-positive cells without peptide pulsing. The discrepancy in T-cell activation between peptide-pulsed T2 and U266-B1 cells can be partially explained by the TAP deficiency in T2 cells, which likely leads to fewer endogenous peptides being presented on both MHC class I and II molecules, and more MART1 peptide presentation. It’s noteworthy that, despite a consistent induction of eGFP across all concentrations of pulsed peptides, increasing ratios of APCs relative to 2D3^TCR/dCas9^ cells resulted in a more pronounced induction of eGFP positive cells (**Supplementary Figure 1**).

We subsequently evaluated TCR activation in co-culture with two HLA-A*02-restricted melanoma cell lines, MALME-3M and SK-MEL-5, which we first engineered to express the dCas9-KRAB-MeCP2 transcriptional repression system. We will further refer to these cells as MALME-3M^dCas9^ and SK-MEL-5^dCas9^. Co-culture with MALME-3M^dCas9^ cells, which exhibit a low baseline expression of PD-L1, resulted in 39.6% (95% CI 39.3%–40.0%) eGFP-positive 2D3^TCR/dCas9^ cells. The addition of nivolumab slightly enhanced T-cell activation, increasing the percentage of eGFP-positive cells to 46.0% (95% CI 45.4%–46.6%). As expected, pre-treatment of MALME-3M^dCas9^ cells with IFN-γ induced PD-L1 expression (22), leading to a marked reduction in T-cell activation, with eGFP-positive cells decreasing to 12.0% (95% CI 11.4%–12.6%). This effect could be rescued by the addition of nivolumab, restoring eGFP expression to approximately 43.0% (95% CI 42.4%–43.6%).

In contrast to MALME-3M^dCas9^, co-culture of 2D3^TCR/dCas9^ with SK-MEL-5^dCas9^ cells resulted in only 16.2% (95% CI 15.8%–16.6%) eGFP-positive T-cells. As SK-MEL-5^dCas9^ has barely detectable levels of PD-L1, nivolumab treatment did not impact T-cell activation. Of note, IFN-γ treatment did not induce PD-L1 expression in SK-MEL-5^dCas9^ cells but did lead to an increase in T-cell activation to 27.3% (95% CI 26.6%–28.0%). No significant change was observed with the subsequent addition of nivolumab following IFN-γ treatment (**Figure 1C**, right). Together, these results demonstrate that SK-MEL-5^dCas9^ cells have a lower immunogenicity compared to MALME-3M^dCas9^ cells, suggesting the presence of one or multiple immune evasion mechanisms in SK-MEL-5^dCas9^ cells.

### 3.2 Comparative immune profiling of SK-MEL-5^dCas9^ and MALME-3M^dCas9^ cell lines

**Figure 2:**
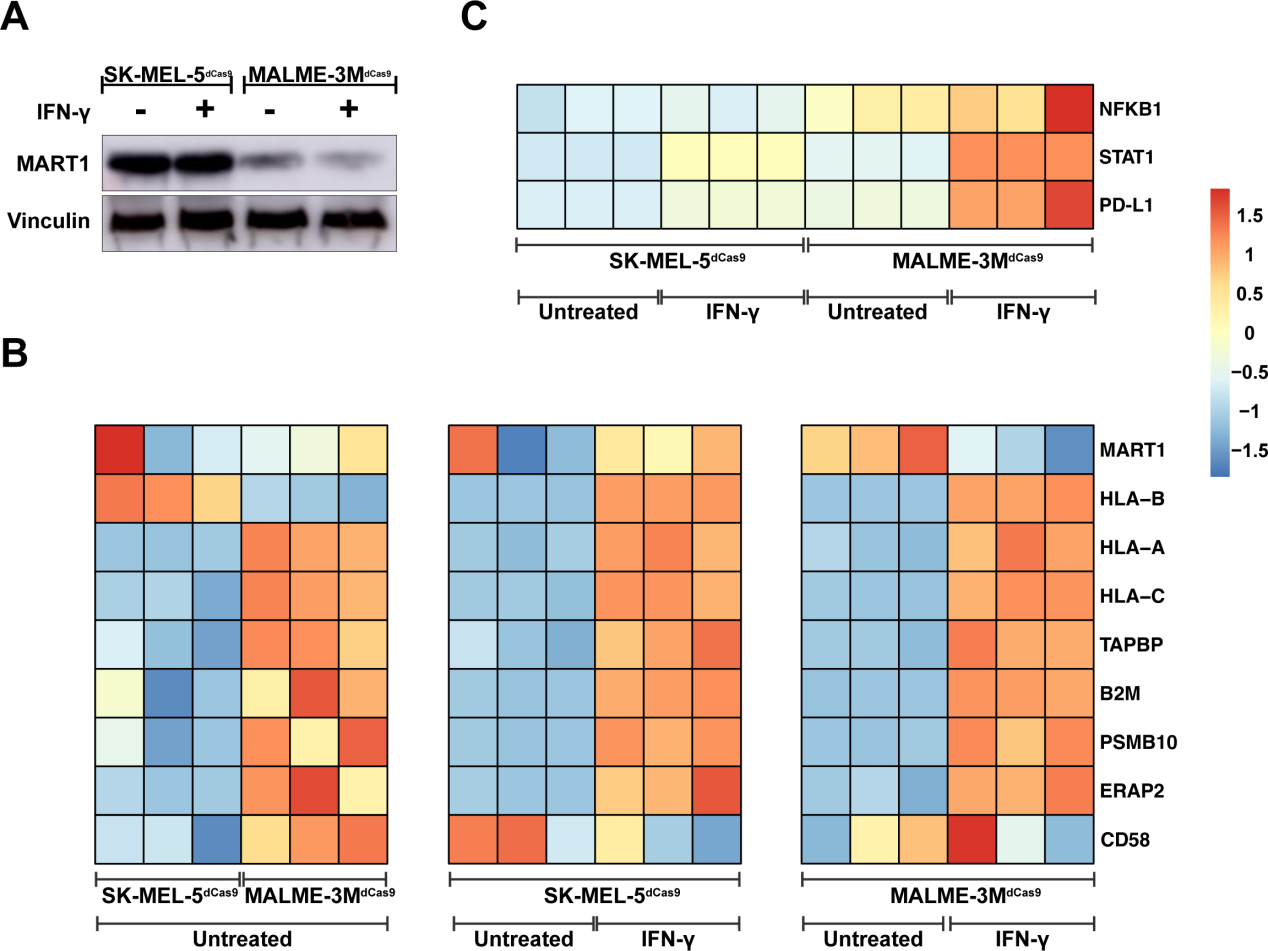
Immune Modulation Landscape and Mechanistic Insights from Melanoma Cell Lines. **(A)** Western blot analysis demonstrating MART1 protein levels in SK-MEL-5^dCas9^ and MALME-3M^dCas9^ melanoma cell lines with and without IFN-γ treatment, revealing higher MART1 expression in SK-MEL-5 cells. **(B)** Heatmap comparison of gene expression profiles, illustrating downregulation of MHC class I pathway genes in untreated SK-MEL-5^dCas9^ cells versus MALME-3M^dCas9^. Post IFN-γ treatment, a heatmap indicates upregulation of MHC class I-associated proteins in both cell lines. **(C)** Post IFN-γ treatment, MALME-3M^dCas9^ cells show an upregulation of the PD-L1 pathway, pointing to a PD-1/PD-L1-mediated reduction in T-cell activation. In contrast, SK-MEL-5^dCas9^ cells exhibit negligible PD-L1 pathway upregulation, suggesting a different mechanism for immune modulation. The color intensity in the heatmap corresponds to the Z-score of normalized counts across rows.

To further investigate the differential immune modulation by both melanoma cell lines, we first quantified protein levels of the MART1 antigen to assess if both cell lines had comparable expression. Western blot (**Figure 2A**) indicated that MART1 protein levels in SK-MEL-5^dCas9^ were even higher compared to MALME-3M^dCas9^, indicating that MART1 expression does not correlate to the T-cell activation capacity of both cell lines. We then performed RNA-sequencing of SK-MEL-5^dCas9^ and MALME-3M^dCas9^ cell lines in the presence or absence of IFN-γ. Sequencing data revealed that SK-MEL-5^dCas9^ cells have reduced expression of MHC class I antigen presentation components relative to MALME-3M^dCas9^ (**Figure 2B**), likely contributing to MALME-3M^dCas9^’s higher T-cell activation rates. Additionally, elevated CD58 expression in MALME-3M^dCas9^ may enhance T-cell engagement and activation *via* CD2 interaction. IFN-γ pretreatment led to upregulated MHC class I-associated proteins in both cell lines. Notably, there was an induction of PD-L1 pathway genes in MALME-3M^dCas9^ (**Figure 2C**), implying a PD-1/PD-L1-mediated reduction in T-cell activation. In contrast, SK-MEL-5^dCas9^ did not exhibit such strong upregulation.

**Figure 3:**
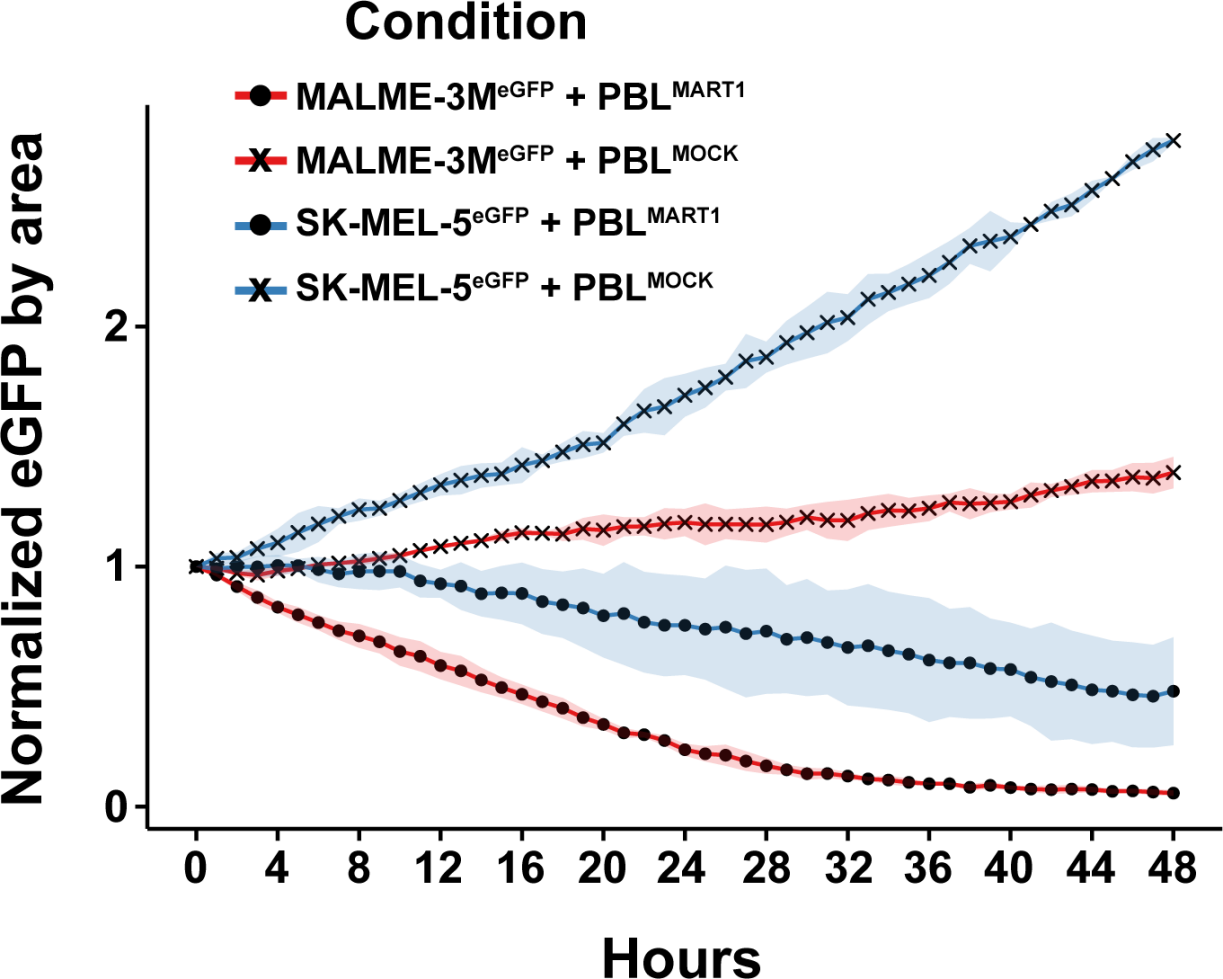
Cytotoxic response of melanoma cell lines to bulk PBL T cell co-culture over 48 hours. Cytotoxic effects of MART1-primed and mock-primed bulk PBL T cells on two melanoma cell lines, MALME-3M^dCas9/eGFP^ and SK-MEL-5^dCas9/eGFP^. The plot shows the normalized eGFP fluorescence intensity, indicative of cell viability, across a 48-hour period.

To validate the observations from our 2D3^TCR/dCas9^ co-culture model, we assessed the cytotoxic effects of bulk PBL T cells, both MART1-primed and mock-primed, against the engineered melanoma cell lines MALME-3M^dCas9/eGFP^ and SK-MEL-5^dCas9/eGFP^. Both cell lines were modified to express eGFP, allowing real-time monitoring of cell viability using the IncuCyte live-cell imaging system over a 48-hour period. In co-cultures with MART1-primed PBL T cells, MALME-3M^dCas9/eGFP^ cells exhibited a substantial decrease in eGFP fluorescence, with a 41% reduction noted within the first 12 hours and reaching up to 80% by 24 hours, reflecting effective antigen-specific T-cell-mediated killing (**Figure 3**). Conversely, when co-cultured with mock-primed PBL T cells, eGFP fluorescence in MALME-3M^dCas9/eGFP^ cells demonstrated an increase—8% in the first 12 hours and 18% after 24 hours—suggesting minimal non-specific cytotoxic effects (**Figure 3**). On the other hand, SK-MEL-5^dCas9/eGFP^ cells demonstrated only a modest decrease in eGFP fluorescence when co-cultured with MART1-primed PBL T cells, with a mere 8% reduction after 12 hours and 25% after 24 hours. This indicates a markedly reduced cytotoxic response compared to that observed with MALME-3M^dCas9/eGFP^ cells (**Figure 3**). Interestingly, when SK-MEL-5^dCas9/eGFP^ cells were co-cultured with mock-primed PBL T cells, there was no visible reduction in cell viability. Instead, these cells exhibited an increase in eGFP intensity, growing by 34% after 12 hours and 71% after 24 hours, indicating a lack of effective T-cell engagement and cytotoxic activity (**Figure 3**). Together, these results confirm the observations with the 2D3^TCR/dCas9^ cells and underscore the value of the model system to study mechanisms of immune evasion.

### 3.3 Validation of dCas9-KRAB-MeCP2 expression and functionality

**Figure 4:**
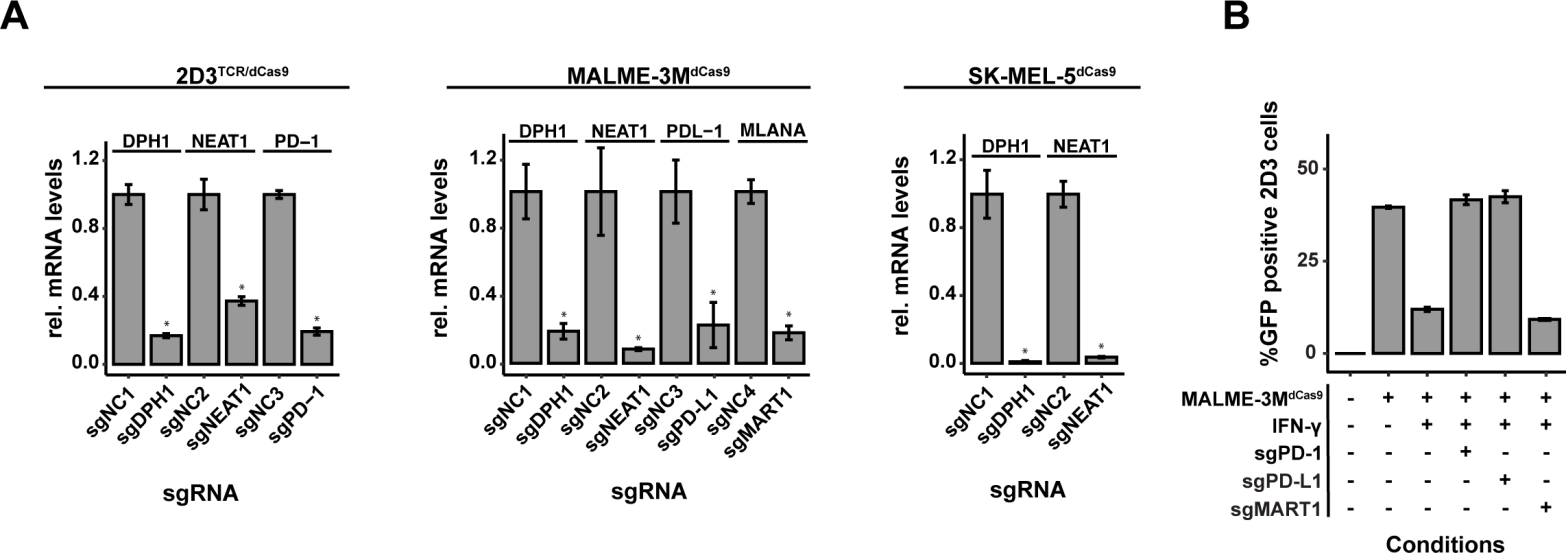
Assessment of dCas9-KRAB-MeCP2 Expression and Gene Targeting Efficacy. **(A)** Quantitative evaluation of gene knockdown efficiency in MALME-3M, SK-MEL-5, and 2D3^TCR/dCas9^ cell lines. Knockdown achieved for all evaluated genes confirmed the precision of CRISPRi-mediated gene silencing. **(B)** Functional validation of the disrupted PD-1/PD-L1 pathway and MART1 in co-cultures of 2D3^TCR/dCas9^ T-cells and MALME-3M melanoma cells. Disruption in either cell type restored T-cell activation, despite significant when IFN-γ was used to induce PD-L1 expression. MART1 knockdown further reduced T-cell activation. All data shown represent the mean values of triplicate measurements, with error bars indicating standard deviation. Statistical significance is denoted by asterisks: *p<0.05 (t-test with Benjamini-Hochberg multiple testing correction).

The integration and functionality of the CRISPRi system in 2D3^TCR/dCas9^, MALME-3M^dCas9^, and SK-MEL-5^dCas9^ cells was verified through different experiments. Western blot (**Supplementary Figure 3**) confirmed the expression of the dCas9-KRAB-MeCP2 protein in all cell lines. Lentiviral delivery of sgRNAs targeting NEAT1 and DPH1 induced a significant knockdown of NEAT1 and DPH1 mRNA expression compared to a scrambled control sgRNA in all cell lines of the co-culture system (**Figure 4A**), confirming functionality of the CRISPRi system. Furthermore, we could demonstrate significant knockdown of PD-1 in 2D3^TCR/dCas9^ cells and PD-L1 in IFN-γ treated MALME-3M^dCas9^ cells upon delivery of purposely designed sgRNAs. CRISPRi-mediated knockdown of PD-1 or PD-L1 in 2D3^TCR/dCas9^ or IFN-γ treated MALME-3M^dCas9^ cells respectively, completely restored T-cell activation in the co-culture setting, to the same extent as treatment with nivolumab (**Figure 4B**). Conversely, knockdown of the MART1 antigen in MALME-3M^dCas9^ cells further reduced T-cell activation (**Figure 4B**). Together, these results demonstrate the potential of this model system to identify modulators of tumor immunogenicity.

### 3.4 Setup of a scalable arrayed sgRNA delivery workflow

**Figure 5:**
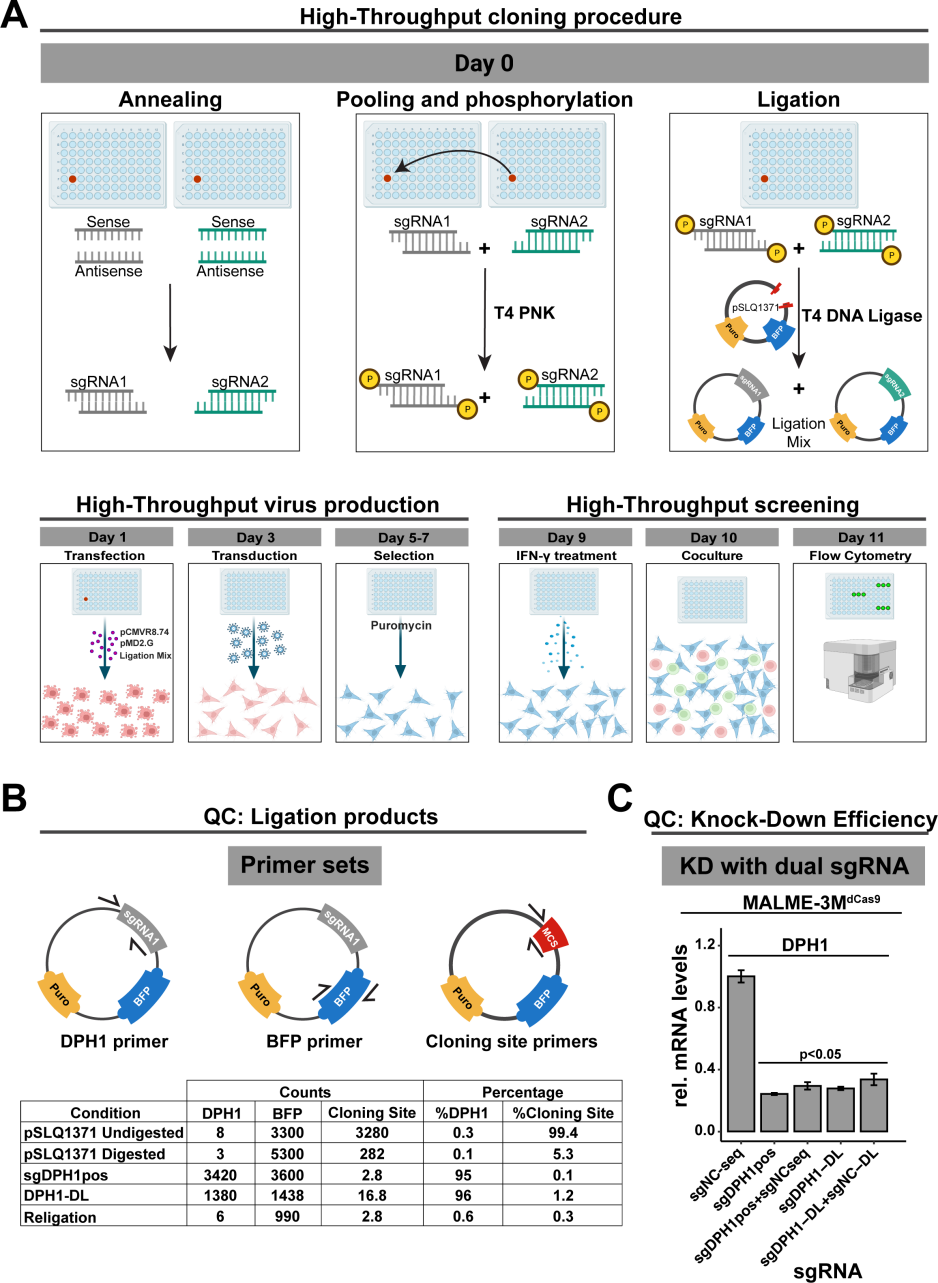
High-Throughput Screening and Validation Workflow for Gene Silencing in Melanoma and T-Cell Co-Culture. **(A)** High-throughput screening workflow for dual sgRNA in the MALME-3M ^dCas9^/2D3^TCR/dCas9^co-culture system. Starting with oligonucleotide annealing and ligation into a carrier vector, the process streamlines sgRNA library creation without bacterial transformation. Lentivirus produced in HEK293T/17 cells is used to infect MALME-3M ^dCas9^ cells, which, after puromycin selection and IFN-γ treatment, are co-cultured with 2D3^TCR/dCas9^cells. eGFP expression is quantified *via* Fortessa-X20 cytometry **(B)** Assessment of ligation efficiency. Droplet digital PCR quantifies ligation using primers for the cloning site, DPH1 gene, and BFP. Results show a 96% success rate for the DPH1 insert, on par with traditional cloning methods. **(C)** Verification of gene silencing efficiency with dual sgRNA constructs. qPCR confirms that adding a non-functional sgRNA does not affect the knockdown efficiency of the target gene. Our high-throughput sgRNA constructs achieve comparable DPH1 knockdown to that of constructs created by conventional cloning. All data shown represent the mean values of triplicate measurements, with error bars indicating standard deviation. Statistical significance is denoted by asterisks: *p<0.05 (t-test with Benjamini-Hochberg multiple testing correction).

To enable a more systematic interrogation of candidate modulators of tumor immunogenicity, we next developed a procedure for direct ligation of spacer in a target vector and lentiviral sgRNA delivery that skips bacterial transformation (**Figure 5A**). To maximize the chances for a successful knockdown of a target gene, we aimed to combine 2 target-specific sgRNAs in a single lentiviral delivery. Sense and antisense oligonucleotides containing the spacer sequence and nucleotide overhangs for cloning were annealed, pooled (pools of 2 spacers) and phosphorylated. Annealed oligos were then ligated at an optimized ratio in a dephosphorylated, double-digested carrier vector, generating a dual population of sgRNA constructs that can be co-transfected with packaging vectors in HEK293T/17 cells for virus production.

We first evaluated the efficiency of our direct ligation method using three digital PCR assays: one targeting the original insert at the cloning site, a DPH1 spacer-specific primer to confirm ligation, and a BFP primer to quantify the vector backbone (**Figure 5B**). To assess vector religation, we included the double-digested pSLQ1371 vector digested but without any insert, referred to as “*Religation*”. Additionally, to evaluate digestion efficiency, we prepared a sample of digested pSLQ1371 in a ligation reaction without T4 DNA ligase, labeled as *‘pSLQ1371 digested*”. A vector with the DPH1 spacer insert acquired through the traditional bacterial cloning and sequencing served as a positive control (*sgDPH1pos*), alongside undigested pSLQ1371 to measure baseline cloning site (*pSLQ1371 undigested*). Upon direct ligation of the DPH1 spacer (*DPH1-DL*), only 1.2% of total vector copies contained the original cloning site suggesting that the phosphatase treatment of the restricted vector effectively prevents vector religation. The majority of vector copies (96%) contained the DPH1 spacer insert, which is similar to what we observed in the positive control sgDPH1pos vector (95%). These results suggest efficient crRNA ligation in a restricted target vector without bacterial transformation. Lentiviral transduction of MALME-3M^dCas9^ cells with the sgDPH1-DL vector resulted in a significant 3.6-fold DPH1 knockdown compared to a negative control sgRNA vector sgNC-DL (**Figure 5C**). Knockdown induced by the sgDPH1-DL vector was similar to that of the sgDPH1pos vector.

To verify that knockdown efficiency is not impacted when combining two spacers in a direct ligation reaction, we combined the NC spacer with the DPH1 spacer in a single reaction to generate a sgDPH1-DL:sgNC-DL mixture for lentiviral transduction. We observed a significant reduction in DPH1 expression that was consistent with the reduction observed with sgDPH1-DL alone, indicating that the presence of an additional crRNA in the ligation reaction does not impact the knockdown efficiency. Similar results were observed when combining a sgDPH1pos and sgNCseq vector, further highlighting the robustness of our direct spacer ligation and lentiviral production approach.

### 3.5 Arrayed perturbation of modulators of tumor immunogenicity

**Figure 6:**
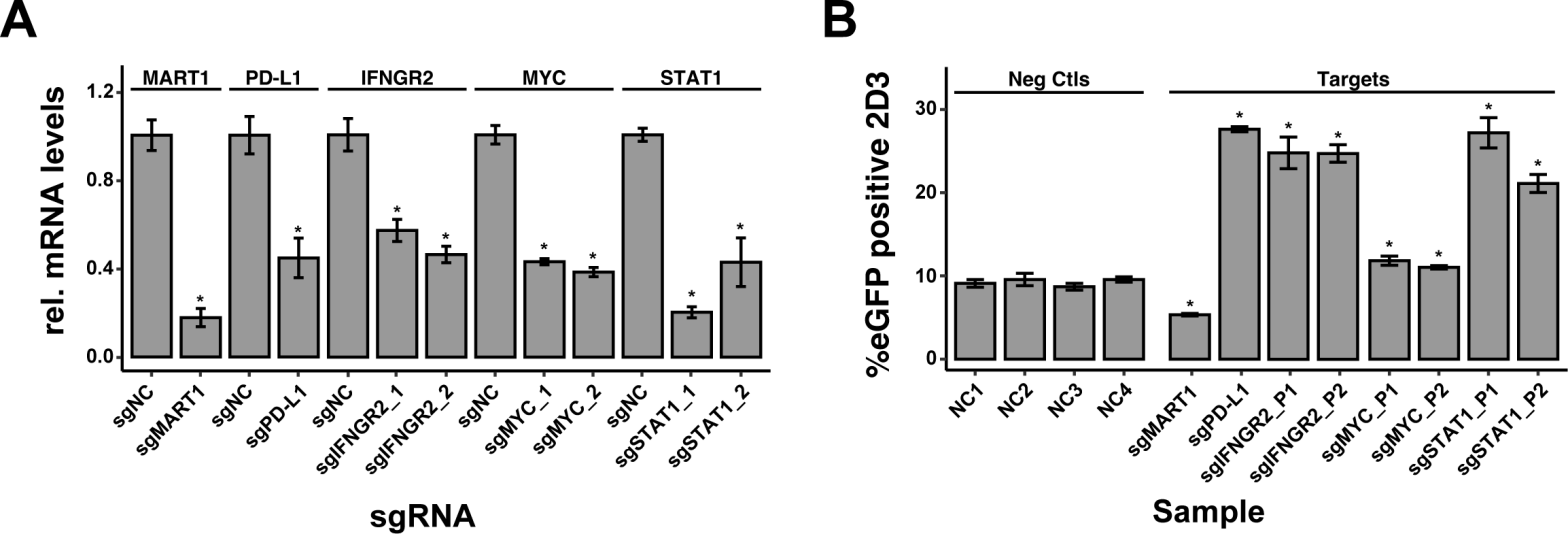
Impact of sgRNA-mediated Knockdown on T-cell Activation in MALME-3M Cells. **(A)** Knockdown efficiency of sgRNA-mediated gene silencing in MALME-3M^dCas9^ cells, as measured by quantitative PCR. The bar graph presents the relative normalized expression levels of five targeted genes: MART1, PD-L1, IFNGR2, STAT1, and MYC, in comparison to non-targeting controls (sgNCs). **(B)** Percentage of eGFP-positive 2D3^TCR/dCas^ T-cells, indicative of T-cell activation levels following sgRNA-mediated knockdown of MART1, PD-L1, IFNGR2, STAT1, and MYC genes in MALME-3M^dCas^ target cells. Positive controls MART1 and PD-L1 knockdowns, conducted with single sgRNA guides, resulted in expected modulation of T-cell activation affirming the arrayed screen’s ability to discern positive and negative modulators of T-cell activation. For MYC, STAT1, and IFNGR2, two pairs of sgRNAs were used to ensure knockdown efficiency. The genetic perturbation of these genes was consistent with their known roles, promoting T-cell activation and thereby validating the sgRNA design and highlighting this method’s potential in investigating gene function in immune response regulation. We used four diverse negative control sgRNAs (sgNC) in the screening to ensure a robust baseline. All data shown represent the mean values of triplicate measurements, with error bars indicating standard deviation. Statistical significance is denoted by asterisks: *p<0.05 (t-test with Benjamini-Hochberg multiple testing correction), calculated based on all combined sgNC.

We next applied our arrayed sgRNA delivery workflow to modulate the expression of genes known to impact tumor immunogenicity. We selected three genes known to modulate tumor immunogenicity (IFNGR2, STAT1 and MYC) for which 2 sgRNA pools were produced as described before. sgRNAs against PD-L1 and MART1 were included as positive controls. For each target, both sgRNA pools induced a significant knockdown of target gene expression upon lentiviral delivery in MALME-3M^dCas9^ cells (**Figure 6A**). To assess the impact of target knockdown on T-cell activation, transduced MALME-3M^dCas9^ cells were treated with IFN-γ and co-cultured with 2D3^TCR/dCas9^ cells. MART1 or PD-L1 knockdown resulted in a significant reduction or increase of eGFP positive 2D3^TCR/dCas9^ cells respectively (**Figure 6B**, left). For IFNGR2, STAT1 and MYC, both sgRNA pools significantly increased the number of eGFP positive 2D3^TCR/dCas9^ cells (**Figure 6B**, right), in line with their role as positive regulators of PD-L1 expression in tumor cells (22, 23). These results further demonstrate the value of the co-culture model system and arrayed sgRNA delivery workflow to identify genes involved in regulating tumor immunogenicity and unravel mechanisms of tumor immune evasion.

## 4 Discussion

We present a tumor cell:T-cell co-culture model system to study modulators of tumor immune evasion through arrayed lentiviral delivery of sgRNAs. We identified 2 melanoma cell lines, MALME-3M and SK-MEL-5, that differentially activate T-cells, implying one or multiple immune evasion strategies that can be investigated mechanistically. In MALME-3M cells, PD-1/PD-L1 interactions prominently mediated immune suppression, as evidenced by the restoration of T-cell activation upon the addition of nivolumab or genetic perturbation of genes in the PD-1/PD-L1 axis. Conversely, the SK-MEL-5 cell line demonstrated immune evasion mechanisms not reliant on PD-1/PD-L1 interactions. This was underscored by the negligible PD-L1 expression, unaffected by IFN-γ exposure, as confirmed through both RNA-sequencing and Western blot analyses. Consequently, nivolumab treatment had no effect. RNA-sequencing of both cell lines revealed impaired antigen presentation and reduced expression of CD58 in SK-MEL-5 cells.

Our bulk PBL T-cell cytotoxicity assay demonstrated that MALME-3M^dCas9/eGFP^ cells were significantly more susceptible to lysis by MART1-primed PBL T cells compared to SK-MEL-5^dCas9/eGFP^ cells, indicating that MALME-3M cells are more receptive to T-cell-mediated killing. This heightened susceptibility in MALME-3M cells can be attributed to their higher expression of MHC class I molecules, which facilitates better antigen presentation and stronger activation of T cells, as evidenced by our 2D3 T-cell model. Additionally, MALME-3M cells exhibit higher levels of co-stimulatory molecules such as CD58, enhancing T-cell activation through interactions with CD2 on T cells. While PBL T cells secrete IFN-γ during the cytotoxic response—which can induce PD-L1 expression on tumor cells—the exposure time and concentration in our assay may not be sufficient for PD-L1 levels to reach an inhibitory threshold before the tumor cells are eliminated. Moreover, PD-L1 expression alone may not be enough to prevent T-cell-mediated cytotoxicity in the presence of robust antigen presentation and co-stimulatory signaling.

The incorporation of the dCas9-KRAB-MeCP2 machinery in combination with a novel workflow for high-throughput spacer ligation and lentiviral delivery enables the exploration of modulators of tumor-immune dynamics. These adaptations further refine previous efforts aimed at establishing co-culture models to study these dynamics (14, 15). This capability is demonstrated by the successful knockdown of strategically selected known modulators of tumor immune evasion validated *in vivo* such as PD-L1 (24–26), IFNGR2 (27, 28), STAT1 (29–31), MART-1 (32, 33), and MYC (34–36). Their knockdown resulted in quantifiable impacts on TCR signaling in the 2D3^TCR/dCas9^ NFAT-driven eGFP reporter cell line. By targeting genes with established roles in tumor immunity from *in vivo* studies, we further demonstrate the applicability and robustness of our model system. Moreover, our model demonstrates robustness, with stable readout across multiple experiments; for instance, knockdown of MART1 in MALME-3M cells was consistent between different experiments, as illustrated in **Supplementary Figure 3**.

While our study highlights the capabilities of our model system and the accompanying workflow for genetic perturbation to reveal key modulators of tumor-immune evasion, it also showcases the potential for broader applications. Although the *in vitro* nature of our co-culture system does not capture every aspect of the tumor microenvironment found *in vivo*, it provides a foundational platform for initial observations. These should be validated through extended studies in animal models and clinical samples. Additionally, exploring the interplay with other immune cells, such as regulatory T-cells (37) and dendritic cells, could offer a more comprehensive understanding of tumor-immune dynamics. Furthermore, our model system is amenable to further refinement by integrating a variety of TCR constructs beyond MART1. This could align the model more closely with the diversity of antigens found across different cancer types, substantially widening the scope of our system. In addition, our model distinguishes itself by offering precise and scalable manipulation of gene expression and a direct, quantifiable measure of T-cell response, facilitating a deeper understanding of the mechanisms underpinning immune evasion and T-cell activation.

## Data Availability

The original contributions presented in the study are publicly available. This data can be found here: GEO with accession number GSE269453, https://www.ncbi.nlm.nih.gov/geo/query/acc.cgi?acc=GSE269453.

## Supplementary material

Supplementary Figure 1 Activation of 2D3TCR/dCas9 Cells in Response to Peptide-Pulsed APC Co-Culture. **(A)** Percentage of GFP-positive 2D3TCR/dCas9 cells following co-culture with peptide-pulsed U266B1 cells. U266B1 cells were pulsed with varying concentrations (5, 25, 50 μg/mL) of MART1 antigen peptide. Co-culture ratios (2D3TCR/dCas9 to U266B1) included 100:1, 10:1, 3:1, 1:1, and 1:2. **(B)** Percentage of GFP-positive 2D3TCR/dCas9 cells following co-culture with peptide-pulsed T2 cells. T2 cells were pulsed with MART1 antigen peptide at concentrations of 1, 5, and 25 μg/mL. Co-culture ratios (2D3TCR/dCas9 to T2) mirrored those used with U266B1.For both cell lines increased APC ratios correlate with elevated GFP expression, reflecting enhanced T-cell activation. In both experiments, co-cultures were incubated for 24 hours before staining with a CD8a-specific antibody and analysis via FORTESSA-X20 cytometer to quantify eGFP-positive 2D3TCR/dCas9 cells. Control conditions include peptide-untreated co-cultures and monocultures of 2D3TCR/dCas9 cells treated with PMA and Ionomycin as negative and positive controls, respectively. All data shown represent the mean values of triplicate measurements, with error bars indicating standard deviation.

Supplementary Figure 2 T-cell Activation in Co-Culture Assays with Varying Treatments and Ratios of 2D3TCR/dCas9 to MALME-3M Cells. GFP expression levels in 2D3TCR/dCas9 cells indicate activation following 24-hour co-culture with MALME-3M cells at cell ratios ranging from 1:1 to 1:5. Treatments include no treatment, Nivolumab (15 μg/mL), and IFN-γ (200 ng/mL) alone or in combination with Nivolumab. The effects of pre-peptide pulsing MALME-3M cells with 5μg MART1 for 24hs are also shown. Controls include untreated 2D3TCR/dCas9 monocultures, and a positive control using PMA (25 ng/mL) and Ionomycin (1 μg/mL). The response is quantified using the FORTESSA-X20 cytometer to measure eGFP-positive cells, providing insight into the immunomodulatory effects of each treatment under varying co-culture conditions. All data shown represent the mean values of triplicate measurements, with error bars indicating standard deviation.

Supplementary Figure 3
**(A)** This panel shows protein levels obtained via western blot of dCas9-KRAB-MeCP2 in 2D3TCR/dCas9, MALME-3MdCas9, and SK-MEL-5dCas9 cell lines, indicating successful expression of CRISPRi components across different cell types. **(B)** This graph presents the percentage of GFP-positive 2D3 cells when co-cultured with MALME-3MdCas9 cells transduced with sgRNAs targeting non-coding control (sgNC), MART1 (sgMART1), and PD-L1 (sgPD-L1) across three experimental dates (2022-03-05, 2022-09-20, and 2023-06-06). The data highlight consistent gene knockdown efficiency and consistency over time. All data shown represent the mean values of triplicate measurements, with error bars indicating standard deviation.
