# Supplementary figures and images for: A co-culture model to study modulators of tumor immune evasion through scalable arrayed CRISPR-interference screens
Source: Front Immunol. 2024 Oct 21;15:1444886. doi: 10.3389/fimmu.2024.1444886 (PMC11532180; doi:10.3389/fimmu.2024.1444886)

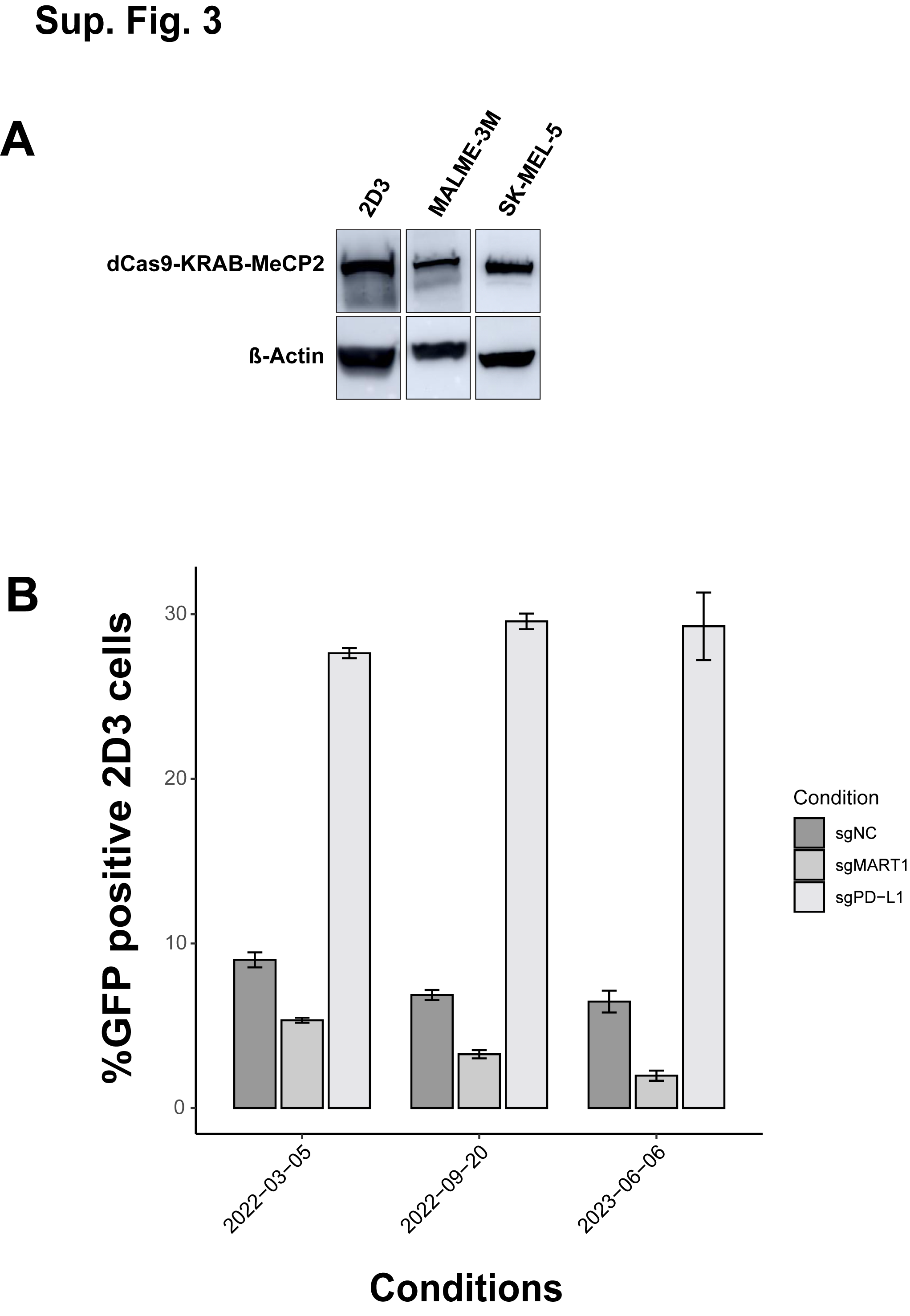

Supplement: Supplementary Figure 1 — Activation of 2D3TCR/dCas9 Cells in Response to Peptide-Pulsed APC Co-Culture. (A) Percentage of GFP-positive 2D3TCR/dCas9 cells following co-culture with peptide-pulsed U266B1 cells. U266B1 cells were pulsed with varying concentrations (5, 25, 50 μg/mL) of MART1 antigen peptide. Co-culture ratios (2D3TCR/dCas9 to U266B1) included 100:1, 10:1, 3:1, 1:1, and 1:2. (B) Percentage of GFP-positive 2D3TCR/dCas9 cells following co-culture with peptide-pulsed T2 cells. T2 cells were pulsed with MART1 antigen peptide at concentrations of 1, 5, and 25 μg/mL. Co-culture ratios (2D3TCR/dCas9 to T2) mirrored those used with U266B1.For both cell lines increased APC ratios correlate with elevated GFP expression, reflecting enhanced T-cell activation. In both experiments, co-cultures were incubated for 24 hours before staining with a CD8a-specific antibody and analysis via FORTESSA-X20 cytometer to quantify eGFP-positive 2D3TCR/dCas9 cells. Control conditions include peptide-untreated co-cultures and monocultures of 2D3TCR/dCas9 cells treated with PMA and Ionomycin as negative and positive controls, respectively. All data shown represent the mean values of triplicate measurements, with error bars indicating standard deviation. [file Image1.tif]

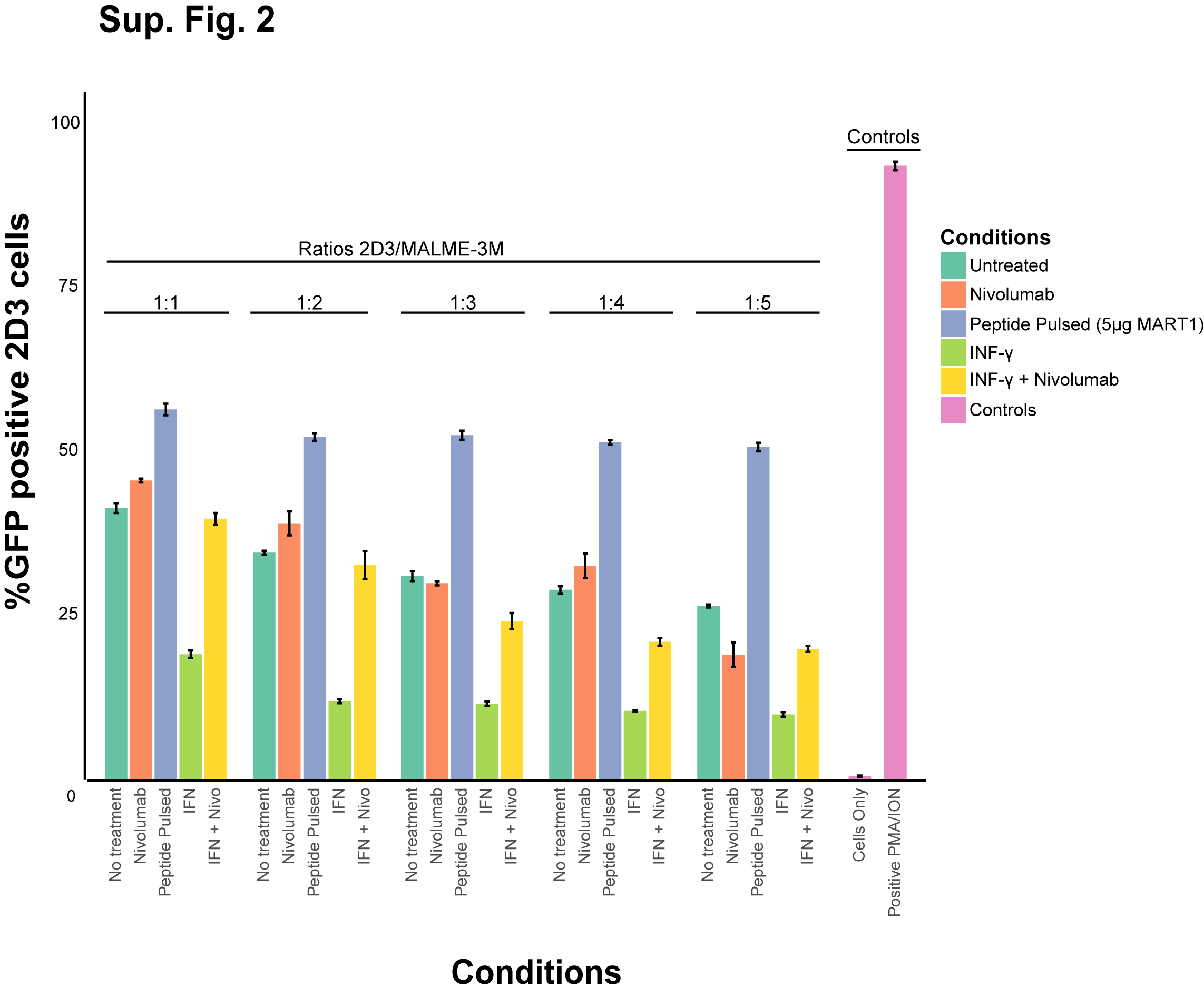

Supplement: Supplementary Figure 2 — T-cell Activation in Co-Culture Assays with Varying Treatments and Ratios of 2D3TCR/dCas9 to MALME-3M Cells. GFP expression levels in 2D3TCR/dCas9 cells indicate activation following 24-hour co-culture with MALME-3M cells at cell ratios ranging from 1:1 to 1:5. Treatments include no treatment, Nivolumab (15 μg/mL), and IFN-γ (200 ng/mL) alone or in combination with Nivolumab. The effects of pre-peptide pulsing MALME-3M cells with 5μg MART1 for 24hs are also shown. Controls include untreated 2D3TCR/dCas9 monocultures, and a positive control using PMA (25 ng/mL) and Ionomycin (1 μg/mL). The response is quantified using the FORTESSA-X20 cytometer to measure eGFP-positive cells, providing insight into the immunomodulatory effects of each treatment under varying co-culture conditions. All data shown represent the mean values of triplicate measurements, with error bars indicating standard deviation. [file Image2.tif]

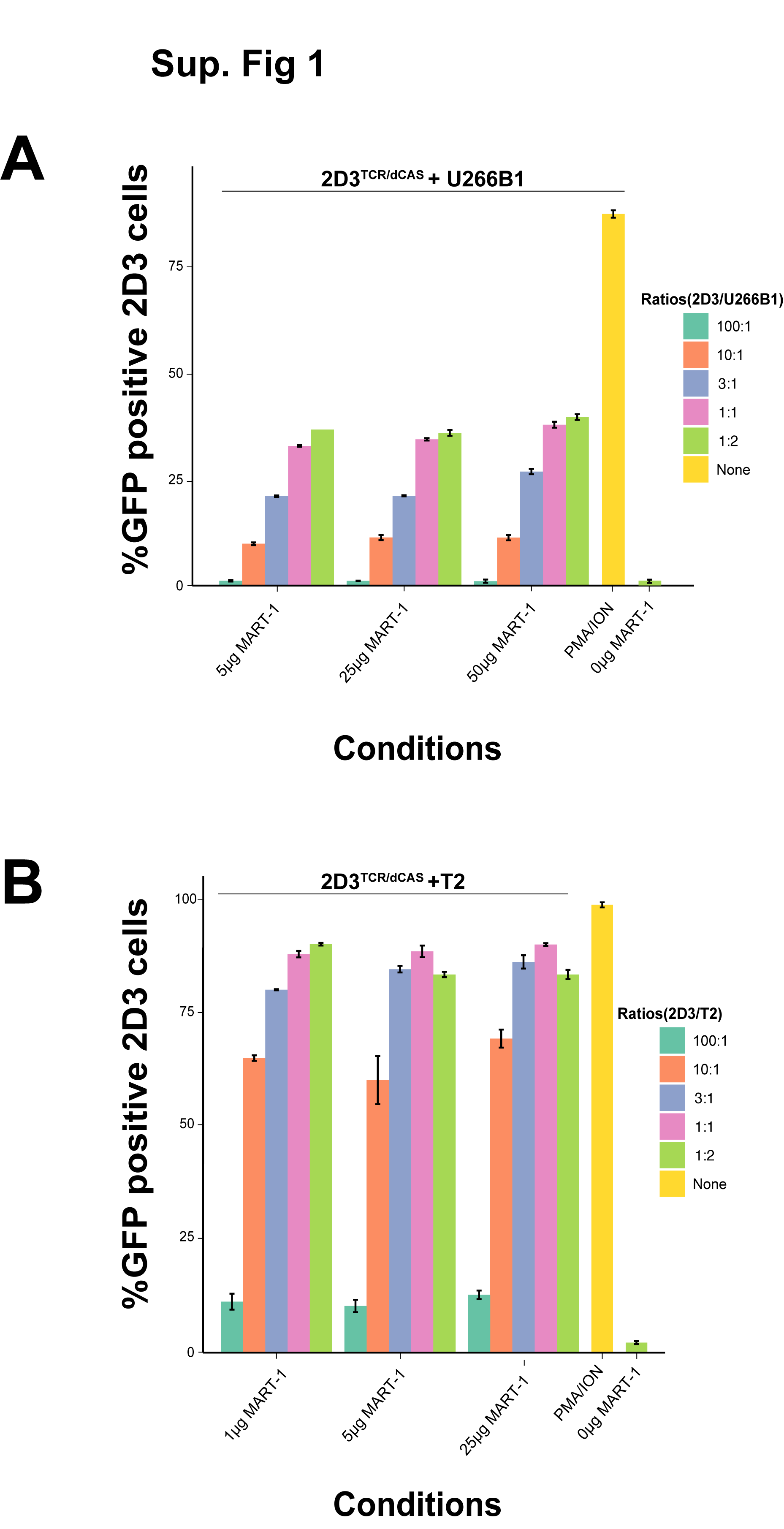

Supplement: Supplementary Figure 3 — (A) This panel shows protein levels obtained via western blot of dCas9-KRAB-MeCP2 in 2D3TCR/dCas9, MALME-3MdCas9, and SK-MEL-5dCas9 cell lines, indicating successful expression of CRISPRi components across different cell types. (B) This graph presents the percentage of GFP-positive 2D3 cells when co-cultured with MALME-3MdCas9 cells transduced with sgRNAs targeting non-coding control (sgNC), MART1 (sgMART1), and PD-L1 (sgPD-L1) across three experimental dates (2022-03-05, 2022-09-20, and 2023-06-06). The data highlight consistent gene knockdown efficiency and consistency over time. All data shown represent the mean values of triplicate measurements, with error bars indicating standard deviation. [file Image3.tif]
